# The role of RICTOR amplification in targeted therapy and drug resistance

**DOI:** 10.1186/s10020-020-0146-6

**Published:** 2020-02-10

**Authors:** Deze Zhao, Man Jiang, Xiaochun Zhang, Helei Hou

**Affiliations:** Department of Medical Oncology, The Affiliated Hospital of Qingdao University, Qingdao University, 16 Jiangsu Road, Qingdao, 266005 China

**Keywords:** RICTOR, TKIs, mTORC2, Drug resistance

## Abstract

The emergence of tyrosine kinase inhibitors (TKIs) has changed the current treatment paradigm and achieved good results in recent decades. However, an increasing number of studies have indicated that the complex network of receptor tyrosine kinase (RTK) co-activation could influence the characteristic phenotypes of cancer and the tumor response to targeted treatments. One of strategies to blocking RTK co-activation is targeting the downstream factors of RTK, such as PI3K-AKT-mTOR pathway. RICTOR, a core component of mTORC2, acts as a key effector molecule of the PI3K-AKT pathway; its amplification is often associated with poor clinical outcomes and resistance to TKIs. Here, we discuss the biology of RICTOR in tumor and the prospects of targeting RICTOR as a complementary therapy to inhibit RTK co-activation.

## Introduction

In the past two decades, cancer treatments have rapidly changed. The model of *precision medicine* was affirmed, and a great deal of targeted drugs have been approved as the first-line treatment for many tumors. Currently, more than 80 molecularly targeted drugs have been developed and applied worldwide, and 47 of these drugs target receptor tyrosine kinase (RTK) activity, including 8 monoclonal antibodies and 39 small-molecule inhibitors (Yamaoka et al. 2018). Although TKIs made a breakthrough in clinical treatments, a large proportion of patients do not benefit from current targeted therapies. One reason is that tumor cells would activate two or more RTKs to maintain signaling networks robustness when facing acute disturbances. The methods to overcome this problem are roughly divided into two categories. The first approach is to simultaneously target multiple RTKs to avoid tumor compensation mechanisms. Another is to identify and target delicate sites located downstream of RTK co-activation networks. In clinical studies, investigators observed that patients with RICTOR amplification had a poor efficacy in taking tyrosine kinase inhibitors; thus, RICTOR was speculated to be involved in resistance to TKIs and has potential to serve as an independent or combined therapeutic target. In this review, we summarize the 1) the biology of RICTOR in tumor including the relationship between RICTOR and RTK and mechanisms of RICTOR in tumor growth, metastasis and drug resistance. 2) preclinical and clinical studies on RICTOR amplification, which provide guidance for designing subsequent clinical trials; and 3) current targeted drugs that inhibit RICTOR.

## The biology of RICTOR in tumor

### RICTOR and RTKs

Receptor tyrosine kinases (RTKs) control basic cellular behaviors such as cell proliferation, apoptosis and migration, and its aberrant activation is regarded as the mechanism driving tumorigenesis and progression (Lemmon and Schlessinger 2010; Robinson et al. 2000). The PI3K/AKT/mTOR pathway, as the major downstream pathway for most RTKs, has become the focus of research on the malignant behavior of tumor cells (Hirsch et al. 2014; Fruman and Rommel 2014). Extensive research has shown that mTORC2 plays an important role in the PI3K-AKT pathway, which could promote cell survival, growth, metabolism and cytoskeletal organization (Saxton and Sabatini 2017; Gan et al. 2012; Li and Gao 2014; Garcia-Martinez and Alessi 2008; Zhang et al. 2010).

RICTOR is a component of the endogenous mTORC2 complex and determines mTORC2 complex stability and integrity (Oh and Jacinto 2011). More than 37 RICTOR phosphorylation sites were identified by mass spectrometry and compared to phosphorylated proteomic data sets. One of the sites, T1135, could be directly phosphorylated by S6K1 and subsequently bind to 14–3-3 proteins, participating in the feedback control of mTORC2 by mTORC1 (Dibble et al. 2009).

### Mechanisms of RICTOR in tumor growth and metastasis

With the in-depth study of RICTOR, researchers found that RICTOR was important for cell proliferation, migration, autophagy and metabolism and could affect cell functions through AKT-dependent and -independent manners.

#### AKT-dependent mechanisms

After the sustained activation of AKT, mTORC2 could affect cell migration and invasion via two coordinated pathways. One of these pathways is the overactivation of AKT, which promotes Rac1 activity by activating the Rac-GEF Tiam1; another such pathway is the suppression of the endogenous Rac1 inhibitor RhoGDI2 through the activation of AKT and PKCα (Morrison Joly et al. 2017). In addition, mTORC2 also regulates glucose metabolism and the synthesis of fatty acids (FA), lipids (glucosylceramide and cardiolipin) and proteins by promoting the release of c-Myc (Oh and Jacinto 2011; Hagiwara et al. 2012; Dang 2012; Plas and Thompson 2005; Huang et al. 2009).

#### AKT-independent mechanisms

In addition, RICTOR could directly activate many downstream molecules. For instance, RICTOR directly phosphorylates the downstream molecule PKCα and inhibits RhoGDI2 (inhibitor of Rac), resulting in the upregulation of RAC1 expression, which enhances chemotaxis and metastatic ability of the cell (Morrison Joly et al. 2017); RICTOR could influence the level of p-c-MET instead of the total level of c-MET to modulate autophagy (Lampada et al. 2017); RICTOR could regulate the expression of HIF-1α and increases the secretion of hypoxia-induced VEGF-A and constitutive IL-8 in response to a hypoxic environment (Schmidt et al. 2017). These processes are impaired by RICTOR elimination and increased by RICTOR overexpression.

### Mechanisms of RICTOR in drug resistance

#### Positive feedback between  RICTOR and AKT

Recent evidence suggests that RICTOR participates in the formation of a positive feedback loop in the AKT pathway. After its activation by upstream RTKs, AKT phosphorylates the mTORC2 subunit SIN1 at T86 and stimulates the activity of mTORC2; subsequently, RICTOR further enhances the phosphorylation of AKT at S473, resulting in the full activation of AKT (Yang et al. 2015; Sarbassov et al. 2005).

Thus, it was proposed that this positive feedback would be significantly enhanced in the amplification of RICTOR, leading to the constant activation of AKT; this process is independent of upstream signals, and ultimately results in tumor progression and drug resistance.

#### Metabolic reprogramming

Metabolic reprogramming is the hallmark of cancer and enables tumor cells to quickly obtain the macromolecular precursors and energy required for growth (Hanahan and Weinberg 2011). Recent research has demonstrated that mTORC2 acts as a central link between glucose metabolism and EGFR-TKI resistance. mTORC2-mediated metabolic reprogramming could lead to the lower spare respiratory capacity (SRC) of cells to cope with glucose deprivation-mediated environmental stress, but this process could be reversed after knockdown of RICTOR (Chiang et al. 2018).

In addition, activated RICTOR could inhibit the phosphorylation of class IIa HDAC and the acetylation of FoxO, subsequently increasing c-Myc levels, thereby regulating cellular metabolism, including the Warburg effect (Masui et al. 2013). More importantly, the increase in glucose and acetate could induce the acetylation of RICTOR via acetyl-CoA and maintain mTORC2 signaling through feedforward activation, causing tumor cells to counteract the TKI-mediated inhibition of upstream signals via AKT-independent pathways (Masui et al. 2015).

Furthermore, mTOR-RICTOR could control cystine uptake and glutathione metabolism by directly phosphorylating xCT, enabling tumor cells to buffer reactive oxygen species (ROS) and transform resources from proliferation to survival processes when the extracellular environment dramatically changes (Gu et al. 2017).

#### Inhibiting apoptosis

NF-κB which is downstream of mTOR, is activated in several types of cancers and is associated with therapy resistance by inhibiting apoptosis (Karin 2006). RICTOR could activate NF-κB and render glioblastoma cells resistant to chemotherapy (Tanaka et al. 2011).

It is worth mentioning that this process does not disappear after blocking AKT phosphorylation but can be reversed by knocking down RICTOR.

**Fig. 1:**
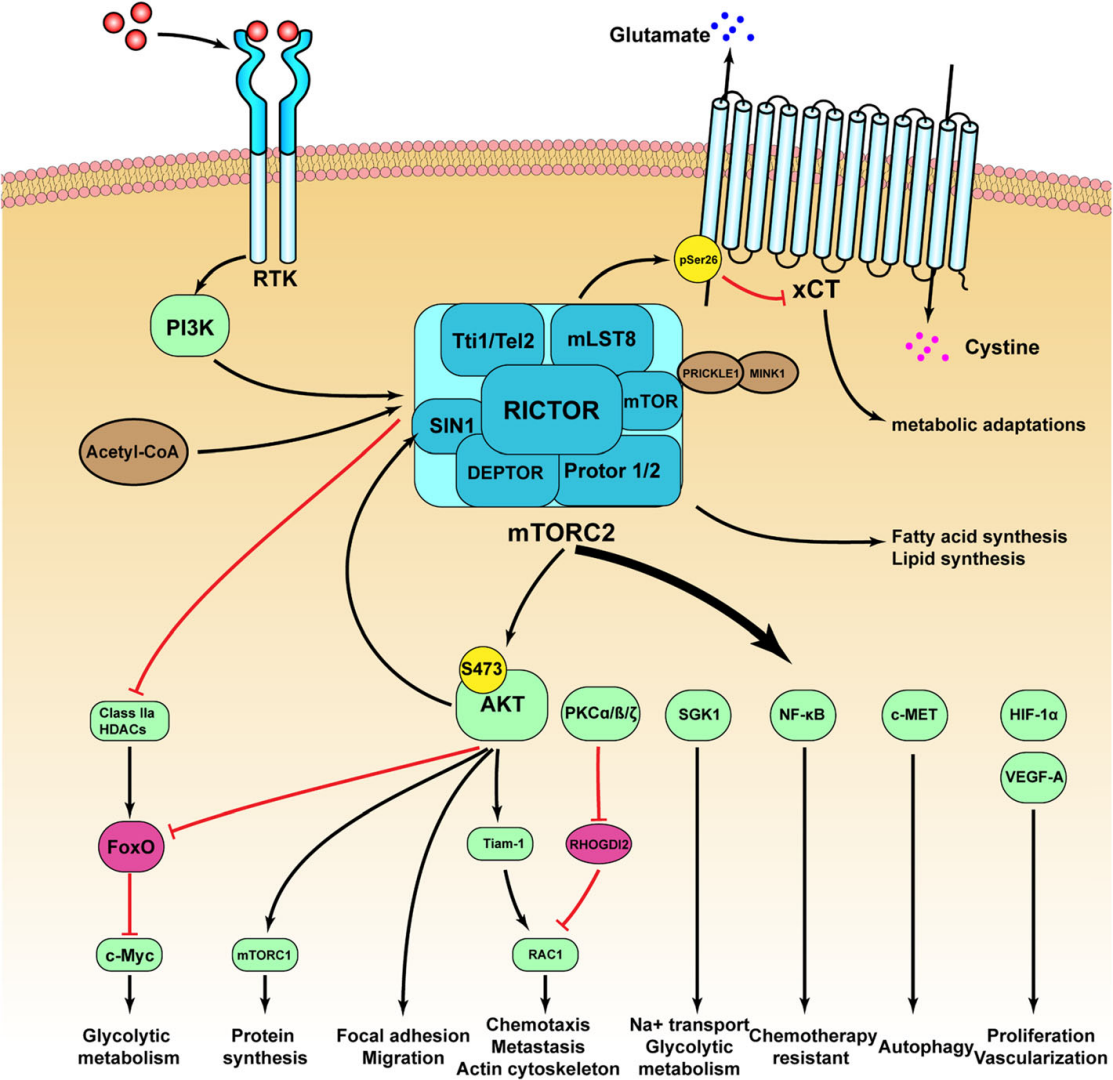
The mechanisms by which RICTOR participating in tumor growth, invasion and drug resistance

Currently, the mechanism by which RICTOR participates in tumor growth, invasion and drug resistance has been shown to be affected by many factors, and the details of these factors are as shown in Fig. 1.

## The prevalence of RICTOR amplification in tumors

**Fig. 2:**
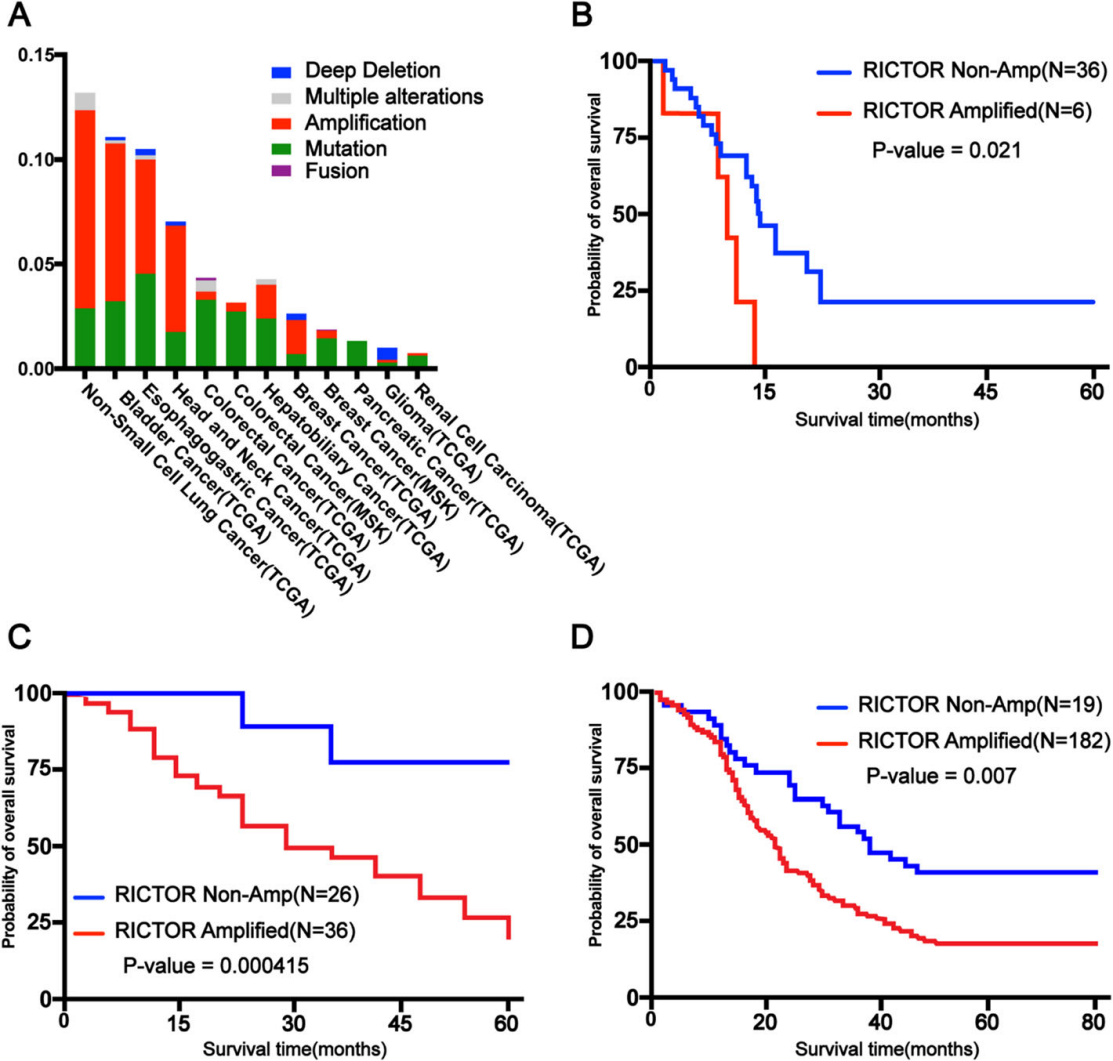
Alteration frequency and clinical outcome of RICTOR amplification in different cancers (**a** Alteration frequency of RICTOR amplification in different cancers. **b** Kaplan-Meier plot on overall survival in SCLC patients with/without RICTOR amplification. **c** Kaplan-Meier plot on overall survival in colorectal cancer patients with/without RICTOR amplification. **d** Kaplan-Meier plot on overall survival in esophageal squamous cell carcinoma patients with/without RICTOR amplification

The mutation rate of *RICTOR* in patients is summarized as follows by querying The Cancer Genome Atlas (TCGA) database (Fig. 2a). The most frequently mutated types of cancer are non-small-cell lung cancer (13.2%, 205/1553), followed by bladder cancer (11.1%, 72/650) and esophageal gastric cancer (10.5%, 104/990) (Cerami et al. 2012; Gao et al. 2013).

Many studies have demonstrated that patients with a high expression of RICTOR in tumor tissue samples have a lower overall survival in cancers, such as small cell lung cancer, colorectal cancer and esophageal squamous cell carcinoma (Fig. 2b-d). A study analyzed patients with SCLC and found that *RICTOR* was the most commonly amplified gene (approximately 14%), and the median overall survival of the *RICTOR* non-amplified and amplified groups was 11.7 months (95% CI: 10.2–18.9) and 7.9 months (95% CI: 1–11.1), respectively (Sakre et al. 2017). Another study reported that the positive expression rate of RICTOR in colorectal cancer tissues was 58.1% (36/62), which correlated with Dukes stage, lymphatic metastasis and prognosis. Patients with a positive expression of RICTOR had a shorter overall survival compared to those with a negative expression, indicating that RICTOR could be used as a prognostic indicator of CRC (Wang et al. 2017). Furthermore, a study analyzed 201 tissue samples of esophageal cancer and found that the percentage of the positive expression of *RICTOR* was 70.6% (142/201). Importantly, the expression was positively correlated with the AJCC stage of patients (*P* = 0.011) and negatively correlated with the survival rate (*P* = 0.007) (Jiang et al. 2017).

In short, *RICTOR* overexpression is associated with tumor malignancy and prognosis, which means RICTOR is a potential drug target.

## Preclinical and clinical studies of RICTOR

### Lung cancer

Currently, lung cancer therapy has shifted from cytotoxic treatments based on physicians’ experiences to personalized precision medicine (Herbst et al. 2018). However, a targetable genomic mutation has not been discovered in nearly half of patients with lung adenocarcinomas (Pao and Hutchinson 2012). In 2015, Cheng evaluated an 18-year-old male never-smoker with lung adenocarcinoma for possible targeted therapy and found that the amplification of *RICTOR* was the only operable genomic alteration. Notably, the patient had more than 18 months of tumor stability after treatment with dual mTOR1 / 2 inhibitors (Cheng et al. 2015). In addition, Dennis (2018) found that *RICTOR* mutations were present in early and advanced lung adenocarcinomas, and the amplification of RICTOR predicted poor overall survival in advanced LUAD patients (OS, HR: 1.73, 95% CI: 1.23–2.42, *p* = 0.0015). Similar results were found in surgically resected LUAD (OS, HR: 1.54, 95% CI: 1.03–2.29, *p* = 0.0337). More importantly, significant enrichment of KRAS/MAPK axis mutations in late and early LUAD had a *RICTOR* mutation, so mTORC1 / 2 and MEK inhibition should be effective treatments for tumors with altered RICTOR / KRAS (Ruder et al. 2018). In conclusion, RICTOR is a feasible novel target for the treatment of lung cancer.

### Bladder Cancer

For 30 years, little progress has been made in the treatment of bladder cancer, and the majority of patients had tumor infiltration at the time of diagnosis (Grayson 2017). Once the tumor invades, the disease-specific survival rate reduces as the pathological stages increase with the current therapeutic interventions. In 2013, Gupta found that the activity of mTORC2 was approximately 5-fold higher in invasive human bladder cancer samples compared to noninvasive samples. Knockdown of *RICTOR* could inhibit the migration and invasion of bladder cancer cells by decreasing the levels of Rac1-GTP and phospho-paxillin (Gupta et al. 2013). Therefore, selectively targeting RICTOR can be a novel strategy for patients with invasive bladder cancer.

### Gastroesophageal cancer

Many studies have shown that the overexpression of mTOR is common in gastric cancer and that p-mTOR is suggested to be an independent prognostic factor for gastric cancer (Yu et al. 2009). To clarify the molecular mechanism and identify novel specific diagnostic markers, Bian (2015) analyzed 396 gastric cancer tissue samples and showed that patients with a positive expression of RICTOR and p-Akt (Ser473) had lower overall survival and recurrence-free survival rates than those with a negative staining for RICTOR. The expression of RICTOR and p-Akt (Ser473) affect lymph node metastasis, TNM staging and WHO classification. Furthermore, the amplification of RICTOR is also associated with tumor size, depth of invasion and tumor thrombosis, whereas p-Akt (Ser473) is associated with distant metastasis (Bian et al. 2015). In 2017, Kim et al. treated a RICTOR-amplified patient-derived cell (PDC) line with selective AKT, mTORC1 and mTORC1/2 inhibitors and found that AZD2014, a dual mTORC1/2 inhibitor, could significantly inhibit the proliferation of PDC and that the knockdown of RICTOR could reverse the sensitivity of PDC to AZD2014. These results suggested that RICTOR amplification was a treatment-related genomic alteration and supported further preclinical and clinical studies of AZD2014 in RICTOR-amplified gastric cancer (Kim et al. 2017a).

### Colorectal cancer

Colorectal cancer (CRC) is the third leading cause of cancer deaths worldwide, and the 5-year survival rate after diagnosis is approximately 65% (Siegel et al. 2017). Recently, autophagy and its relation to drug resistance have become a hot research topic in CRC (Lampada et al. 2017; Shuhua et al. 2015). To determine the specific mechanism of autophagy, Siegel (2017) analyzed 279 colorectal cancer specimens and found that the protein and mRNA expression levels of RICTOR, LC3, MDR-1, Raptor, mTOR and Beclin1 were significantly higher than those of adjacent tissues. Among them, the expression of RICTOR was positively correlated with LC3 (r_s_ = 0.168, *P* < 0.01) and MDR-1 (r_s_ = 0.427, *P* < 0.01) and negatively correlated with RAPTOR (r_s_ = − 0.669, *P* < 0.01). As a result, these researchers indicated that autophagy is tightly correlated with MDR-induced resistance in CRC (Shuhua et al. 2015).

### Hepatocellular carcinoma

Hepatocellular carcinoma (HCC), the most common primary liver cancer, is the leading cause of death in patients with cirrhosis, and its incidence has continued to rise in recent years (Forner et al. 2018). Choline kinase alpha (CHKA), the first enzyme in the Kennedy pathway, is shown to be involved in HCC metastasis and resistance to EGFR-targeted drugs. When studying specific mechanisms, Lin (2017) accidentally discovered that silencing RICTOR abolished CHKA-induced resistance to gefitinib and erlotinib and even increased the sensitivity of cells to drugs (Lin et al. 2017).

Therefore, the selective blocking of RICTOR can be used as a treatment strategy for EGFR-resistant tumors.

### Breast cancer

In recent decades, various novel treatment options for breast cancer (BC) have been developed and have obviously improved the clinical outcomes for patients (Waks and Winer 2019). However, overactive PI3K/Akt/mTOR signaling makes tumor cells avoid cytotoxicity and resist treatment (Miller et al. 2011a; Miller et al. 2011b). In 2016, Meghan et al. found that RICTOR was enriched in HER2-amplified samples and proved that RICTOR-mediated Akt phosphorylation (s473), instead of mTORC1-mediated phosphorylation, could maintain the survival of HER2-amplified breast cancer cells. In addition, RICTOR ablation increased lapatinib-mediated cell death in HER2-amplified breast cancer cells, and similar results occurred with the combined use of lapatinib and PP242 (dual mTORC1/2 inhibition). Subsequently, researchers established a lapatinib-resistant cell model. After the knockdown of RICTOR, the sensitivity of lapatinib-resistant cells to drugs was reversed. These results indicate that the TKI-resistant cells are exquisitely sensitive to RICTOR/mTORC2 targeting and that the combined use of TKIs and dual mTOR inhibitors is an effective therapeutic strategy (Morrison Joly et al. 2016).

Recently, several studies showed that inhibiting mTORC2 while retaining mTORC1 signaling is advisable (Palm et al. 2015; O'Reilly et al. 2006; Rozengurt et al. 2014; Carracedo et al. 2008). Thomas et al. (2018) developed a nanoparticle-based RNAi therapeutic that could effectively silence the mTORC2 cofactor RICTOR. By intratumoral or intravenous delivery, nanomedicine in combination with lapatinib impaired the survival of HER2-amplified breast cancer cells (Werfel et al. 2018).

This study provides a new approach for the selective inhibition of RICTOR and provides motivation for subsequent clinical trials.

### Pancreatic cancer

Pancreatic cancer (PC) is one of the most fatal diseases, with a 5-year overall survival rate of only 9% (Siegel et al. 2019). Different from the increase in the survival rate of other cancers, few advancements have been made in PC (Kamisawa et al. 2016). In 2017, Katharina assessed the correlation between RICTOR in PC samples and the survival of PC patients and found that the expression of RICTOR significantly reduced the survival of PC patients (*p* < 0.0001). Subsequent in vitro and in vivo experiments suggested that RICTOR blockade impaired tumor growth via decreasing the activation of the AGC kinase and decreasing the expression of hypoxia-inducible factor 1-alpha (HIF-1α) and VEGF-A (Schmidt et al. 2017). Another study obtained similar results and further tested the effects of the dual mTORC1/2 inhibitor (AZD2014) on mice with pancreatic tumors. Compared to gemcitabine or AZD2014 alone, AZD2014 combined with gemcitabine significantly prolonged the survival of mice with early-stage tumors (median survival, 280 vs. 147 days). Interestingly, AZD2014 alone, instead of combination therapy, prolonged the survival of mice with late-stage tumors at the start of the treatment (Driscoll et al. 2016).

Detailed mechanisms need to be studied further, but these results propose a new therapeutic strategy for PC.

### Glioma

Glioblastoma (GBM) is one of the most lethal and persistent malignant tumors (Alexander and Cloughesy 2017). Due to the common alteration of *EGFR* and *PTEN* in GBM, many therapeutic strategies have been developed (Molina et al. 2008). However, a phase II trial of EGFR-TKI plus mTOR inhibitor in adults with recurrent glioblastoma failed to gain satisfactory results (Reardon et al. 2010). A possible explanation for these results is the feedback mechanism of the inhibition of mTORC1 stimulating the mitogenic pathways (Julien et al. 2010). Luchman (2014) assessed the effect of the dual mTORC1/2 inhibitor (AZD8055) and found that AZD8055 significantly reduced the viability of glioma cells regardless of their EGFR and PTEN mutation status. Systemic administration of the drug could reduce tumor growth in subcutaneous xenografts but not improve the survival of animals with orthotopic xenografts. One possible reason for this is that the blood-brain barrier prevents sustained intracranial concentrations from reaching a certain amount (Luchman et al. 2014). Other related dual mTORC1/2 inhibitors have also achieved good results in GBM and includePP242 and JR-AB2–011 (Mecca et al. 2018; Benavides-Serrato et al. 2017). These results indicate that mTORC2 inhibition is a feasible strategy for the treatment of GBM.

### Immunity therapy

Recently, the success of checkpoint blockade therapy noticeably inspired research into immune therapy (Sharma and Allison 2015). The detection of immunometabolism emphasizes the importance of cellular metabolism on the biological functions of immune cells (Buck et al. 2017). RICTOR, as the core component of mTORC2, was found to play an important role in regulating immune cells (Zeng 2017).

#### Dendritic cells

Dendritic cells (DCs) are the most effective antigen-presenting cells for initiating the T cell response. In 2015, Raïch-Regué et al. demonstrated that RICTOR ablation increased DC secretion of pro-inflammatory cytokines (IL-6 and IL-23), thereby promoting Th1/Th17 responses and T cell proliferation (Raich-Regue et al. 2015).

These results illustrate a new role of mTOR in DCs and suggest that an mTORC2-selective inhibitor is a potential treatment for immune-mediated inflammation and anti-tumor immunity.

#### CD8^+^ T cells

CD8^+^ T cells are the main effector cells of the immune system, and these cells perceive antigens presented by MHC class I molecules (Godfrey et al. 2018). Several studies have shown that depletion of antigen-specific CD8+ T cells significantly affects the efficacy of immunotherapy (Im et al. 2016; Chang et al. 2014).

In 2015, Kristen et al. used an adoptive transfer model to clarify the role of mTORC2 in the function of CD8+ T cells. The results suggested that RICTOR^- / -^ T cells showed not only strong differentiation into memory cells but also increased responses when stimulated again (Pollizzi et al. 2015).

To further investigate the mechanism of mTORC2 shaping CD8 effector and memory differentiation, Zhang (2016) et al. developed a hybrid mouse model and suggested that the mTORC2-Akt-Foxo1 signaling axis is the crucial regulator of CD8 T cell effector and memory differentiation. Silencing of RICTOR enhanced the reservation of FOXO1 in the nucleus, thereby upregulating the expression of Eomes and Tcf-1, repressing the expression of T-bet and enhancing the mitochondrial spare respiratory capacity and fatty acid oxidation (Zhang et al. 2016).

These studies indicated that the RICTOR blockade could enhance the generation of memory T cells without impairing the effector response. Therefore, selective inhibition of mTORC2 may be an important target for immunotherapy interventions.

## Drugs targeting RICTOR/mTORC2

As early as 1984, the first generation of mTOR inhibitor, rapamycin, was tried for tumor treatment (Eng et al. 1984), and subsequent reports suggested that the combined use of rapamycin and other drugs had great antitumor effect (Bae-Jump et al. 2009; Han et al. 2012; Shafer et al. 2010; Kimura and Huang 2016). However, rapamycin only targeted mTORC1 and showed limited response rates in cancer treatments. Later, second-generation rapamycin derivatives were developed, and it has been proven to have more effective pharmacokinetic properties and better anti-cancer effects in many clinical trials (Zhou and Huang 2012; Motzer et al. 2016). Currently, the second generation of mTOR inhibitors target both mTORC1 and mTORC2 and include 1) ATP-competitive TKI, targeting mTORC1 and mTORC2; 2) dual inhibitors, targeting PI3K along with mTORC1 and mTORC2; and 3) rapamycin, inhibiting mTORC1 and the assembly of mTORC2. More detailed drug information is shown in Table 1. Most of these drugs are in clinical trials, and the combined regimen shows better therapeutic effects than monotherapy based on several results reports.

**Table 1:** Different types of mTORC2 targeted drugs

Drug type	Name	Tumor or cell line	Reference
ATP-competitive mTOR inhibitor	CZ415	Head and neck squamous cell carcinoma (HNSCC), papillary thyroid carcinoma	(Xie et al. 2018; Li et al. 2018)
	Torin2	Adult T-cell leukemia/lymphoma, anaplastic thyroid cancer	(Watanabe et al. 2016; Sadowski et al. 2015)
	MLN0128 (INK128)	Intrahepatic cholangiocarcinoma Neuroblastoma, breast cancer, osteosarcoma	(Zhang et al. 2017; Zhang et al. 2015; Liu et al. 2016)
	PP242	Glioblastoma, ovarian cancer	(Mecca et al. 2018; Kawata et al. 2018; Musa et al. 2016)
	AZD8055	Adult T-cell leukemia (ATL), hepatocellular carcinoma, colon cancer, neuroblastoma	(Kawata et al. 2018; Chen et al. 2018; Xu et al. 2018)
	AZD2014	Diffuse intrinsic pontine glioma (DIPG), gastric cancer, anaplastic thyroid carcinoma (ATC), ovarian cancer	(Kim et al. 2017a; Milosevic et al. 2018; Wong Te Fong et al. 2017; Flannery et al. 2018)
PI3K/mTOR inhibitor	Dactolisib (BEZ235)	Colon cancer, glioblastomas, breast cancer	(Yu et al. 2016; Shi et al. 2018; Torki et al. 2017)
	GSK1059615	Head and neck squamous cell carcinoma (HNSCC)	(Xie et al. 2017a)
	LY3023414	Esophageal Adenocarcinoma	(Zaidi et al. 2017)
	voxtalisib (SAR245409, XL765)	endometrial carcinoma	(Yu et al. 2014; Inaba et al. 2015)
	PQR309	brain tumor or CNS metastasis	(Beaufils et al. 2017)
	gedatolisib (PKI-587, PF05212384)	breast, colon, lung, and glioma carcinoma	(Venkatesan et al. 2010)
	omipalisib (GSK2126458)	breast carcinoma	(Knight et al. 2010)
mTORC1/2 dual inhibitor	CC-223	Hepatocellular carcinoma, colorectal cancer, head and neck squamous cell carcinoma (HNSCC)	(Xie et al. 2017b; Wang et al. 2018a)
	Ku0063794	Hepatocellular carcinoma	(Kim et al. 2017b)
	OSI027	Pancreatic cancer, colon cancer	(Chen et al. 2015; Bhagwat et al. 2011; Zhi et al. 2015)
	RES-529 (Palomid 529)	Osteosarcoma, angiogenesis and vascular permeability, prostate cancer	(Hu et al. 2018; Xue et al. 2008; Gravina et al. 2011)
	WYE-687	Renal carcinoma, acute myeloid leukemia (AML)	(Pan et al. 2017; Cheng et al. 2016)
	WYE-354	Colon cancer, gallbladder cancer	(Wang et al. 2016; Weber et al. 2015)

## Discussion

In 2010, Xu et al. demonstrated that the receptor tyrosine kinase (RTK) coactivation network was an important mechanism promoting tumor development and limiting the lethal effects of targeted drugs (Xu and Huang 2010). Therefore, aiming to determine the individual signaling pathway of an RTK is no longer appropriate, and targeting the downstream pathways of RTKs has become a new strategy to solve this issue (Tan et al. 2017). RICTOR, the core component of the PI3K/Akt pathway, has been shown to be involved in tumor survival and drug resistance. Several preclinical experiments have shown that the single or combined use of mTOR inhibitors can significantly inhibit tumor growth, increase cell sensitivity to TKIs, and even reverse drug resistance (Lin et al. 2017; Zheng et al. 2015).

Currently, relevant clinical trials about an inhibitor of RICTOR (AZD2014) are ongoing (ClinicalTrials.gov Identifier: NCT03061708 and NCT03166904).

As described earlier, nonselective inhibition of the mTOR pathway may cause off-target effects due to the complexity of the PI3K/AKT/mTOR pathway. For example, even in the case of mTORC2 inhibition, blocking mTORC1 would activate negative feedback loops, which causes AKT to reactivate (Fruman and Rommel 2014; Aylett et al. 2016). Therefore, it is necessary to find a specific drug that inhibits RICTOR. Recently, Benavides (2017) identified a small molecule (CID613034) by utilizing a high-throughput yeast two-hybrid screen. This small molecular specifically inhibited the phosphorylation of mTORC2 but had no significant effects on the phosphorylation status of the mTORC1 substrate S6K (Thr-389) and therefore does not activate the mTORC1-dependent negative feedback loop (Benavides-Serrato et al. 2017). In addition, Thomas (2019) reported another effective method; a nanoparticle-based RNAi therapeutic specifically silences the mTORC2 obligate cofactor RICTOR (Werfel et al. 2018).

These selective mTORC2 inhibitors all achieved good results in in vitro and xenograft experiments, providing feasible and efficacious treatments for refractory cancers.

The immunosuppressive activity of rapamycin was first proposed in 1977 (Martel et al. 1977), and its function in the immune system has been increasingly valued since then (Waldner et al. 2016). Preclinical experiments have found that RICTOR regulates the biological functions of various immune cells, and its knockdown would be one of the ways to enhance the efficacy of immunotherapy (Zeng 2017; Pollizzi et al. 2015; Zhang et al. 2016). However, some basic studies have shown that mTOR regulates the homeostasis of immune cells in an interactive manner (Wang et al. 2018b), thus, targeting mTOR in immune cells may destroy the immune tolerance and lead to autoimmune diseases. Autoimmune syndrome is an important factor in the failure of immunotherapy (June et al. 2017).

As a result, when using targeted or adjuvant immunotherapy, doctors need to carefully record the patient status and promptly resolve immune-related adverse events (irAEs) to minimize potential risks. Currently, there are no relevant immune-related trials; however, based on the regulatory functions of RICTOR in different immune cells, it is a promising cellular target for cancer immunotherapy.

## Conclusion

As a key effector molecule of PI3K/AKT/mTOR, RICTOR plays an important role in tumorigenesis and invasion and causes tumor resistance to RTK-TKIs by AKT-dependent and -independent pathways, which seriously limits patients’ benefits from targeted drugs. Therefore, RICTOR is an important potential target for addressing drug resistance issues. Additional studies are needed to elucidate the mechanism of RICTOR and will provide definitive evidence for future clinical trials.

## Data Availability

Data and materials are from article of Pubmed.

## Abbreviations

BC: Breast cancer
CHKA: Choline kinase alpha
CRC: Colorectal cancer
DCs: Dendritic cells
GBM: Glioblastoma
HCC: Hepatocellular carcinoma
irAEs: Immune-related adverse events
NSCLC: Non-small-cell lung cancer
PC: Pancreatic cancer
ROS: Reactive oxygen species
RTK: Receptor tyrosine kinase
SCLC: Small-cell lung cancer
SRC: Spare respiratory capacity
TCGA: The Cancer Genome Atlas
TKIs: Tyrosine kinase inhibitors

## References

[CR1] Alexander BM, Cloughesy TF (2017). Adult Glioblastoma. J Clin Oncol..

[CR2] Aylett CH, Sauer E, Imseng S, Boehringer D, Hall MN, Ban N (2016). Architecture of human mTOR complex 1. Sci..

[CR3] Bae-Jump VL, Zhou C, Boggess JF, Gehrig PA (2009). Synergistic effect of rapamycin and cisplatin in endometrial cancer cells. Cancer..

[CR4] Beaufils F, Cmiljanovic N, Cmiljanovic V, Bohnacker T, Melone A, Marone R (2017). 5-(4,6-Dimorpholino-1,3,5-triazin-2-yl)-4-(trifluoromethyl)pyridin-2-amine (PQR309), a potent, brain-penetrant, orally bioavailable, Pan-class I PI3K/mTOR inhibitor as clinical candidate in oncology. J Med Chem.

[CR5] Benavides-Serrato A, Lee J, Holmes B, Landon KA, Bashir T, Jung ME (2017). Specific blockade of Rictor-mTOR association inhibits mTORC2 activity and is cytotoxic in glioblastoma. PLoS One.

[CR6] Bhagwat SV, Gokhale PC, Crew AP, Cooke A, Yao Y, Mantis C (2011). Preclinical characterization of OSI-027, a potent and selective inhibitor of mTORC1 and mTORC2: distinct from rapamycin. Mol Cancer Ther.

[CR7] Bian Y, Wang Z, Xu J, Zhao W, Cao H, Zhang Z (2015). Elevated Rictor expression is associated with tumor progression and poor prognosis in patients with gastric cancer. Biochem Biophys Res Commun.

[CR8] Buck MD, Sowell RT, Kaech SM, Pearce EL (2017). Metabolic instruction of immunity. Cell..

[CR9] Carracedo A, Ma L, Teruya-Feldstein J, Rojo F, Salmena L, Alimonti A (2008). Inhibition of mTORC1 leads to MAPK pathway activation through a PI3K-dependent feedback loop in human cancer. J Clin Invest.

[CR10] Cerami E, Gao J, Dogrusoz U, Gross BE, Sumer SO, Aksoy BA (2012). The cBio cancer genomics portal: an open platform for exploring multidimensional cancer genomics data. Cancer Discov..

[CR11] Chang JT, Wherry EJ, Goldrath AW (2014). Molecular regulation of effector and memory T cell differentiation. Nat Immunol.

[CR12] Chen B, Xu M, Zhang H, Xu MZ, Wang XJ, Tang QH (2015). The Antipancreatic Cancer activity of OSI-027, a potent and selective inhibitor of mTORC1 and mTORC2. DNA Cell Biol.

[CR13] Chen Y, Lee CH, Tseng BY, Tsai YH, Tsai HW, Yao CL (2018). AZD8055 exerts antitumor effects on Colon Cancer cells by inhibiting mTOR and cell-cycle progression. Anticancer Res.

[CR14] Cheng F, Wang L, Shen Y, Xia J, Chen H, Jiang Y (2016). Preclinical evaluation of WYE-687, a mTOR kinase inhibitor, as a potential anti-acute myeloid leukemia agent. Biochem Biophys Res Commun.

[CR15] Cheng H, Zou Y, Ross JS, Wang K, Liu X, Halmos B (2015). RICTOR amplification defines a novel subset of patients with lung Cancer who may benefit from treatment with mTORC1/2 inhibitors. Cancer Discov.

[CR16] Chiang CT, Demetriou AN, Ung N, Choudhury N, Ghaffarian K, Ruderman DL (2018). mTORC2 contributes to the metabolic reprogramming in EGFR tyrosine-kinase inhibitor resistant cells in non-small cell lung cancer. Cancer Lett.

[CR17] Dang CV (2012). Links between metabolism and cancer. Genes Dev.

[CR18] Dibble CC, Asara JM, Manning BD (2009). Characterization of Rictor phosphorylation sites reveals direct regulation of mTOR complex 2 by S6K1. Mol Cell Biol.

[CR19] Driscoll DR, Karim SA, Sano M, Gay DM, Jacob W, Yu J (2016). mTORC2 signaling drives the development and progression of pancreatic Cancer. Cancer Res.

[CR20] Eng CP, Sehgal SN, Vezina C (1984). Activity of rapamycin (AY-22,989) against transplanted tumors. J Antibiot (Tokyo).

[CR21] Flannery PC, DeSisto JA, Amani V, Venkataraman S, Lemma RT, Prince EW (2018). Preclinical analysis of MTOR complex 1/2 inhibition in diffuse intrinsic pontine glioma. Oncol Rep.

[CR22] Forner A, Reig M, Bruix J (2018). Hepatocellular carcinoma. Lancet (London, England).

[CR23] Fruman DA, Rommel C (2014). PI3K and cancer: lessons, challenges and opportunities. Nat Rev Drug Discov.

[CR24] Gan X, Wang J, Wang C, Sommer E, Kozasa T, Srinivasula S (2012). PRR5L degradation promotes mTORC2-mediated PKC-delta phosphorylation and cell migration downstream of Galpha12. Nat Cell Biol.

[CR25] Gao J, Aksoy BA, Dogrusoz U, Dresdner G, Gross B, Sumer SO (2013). Integrative analysis of complex cancer genomics and clinical profiles using the cBioPortal. Sci Signal.

[CR26] Garcia-Martinez JM, Alessi DR (2008). mTOR complex 2 (mTORC2) controls hydrophobic motif phosphorylation and activation of serum- and glucocorticoid-induced protein kinase 1 (SGK1). Biochem J.

[CR27] Godfrey DI, Le Nours J, Andrews DM, Uldrich AP, Rossjohn J (2018). Unconventional T cell targets for Cancer immunotherapy. Immun..

[CR28] Gravina GL, Marampon F, Petini F, Biordi L, Sherris D, Jannini EA (2011). The TORC1/TORC2 inhibitor, Palomid 529, reduces tumor growth and sensitizes to docetaxel and cisplatin in aggressive and hormone-refractory prostate cancer cells. Endocr Relat Cancer.

[CR29] Grayson M (2017). Bladder cancer. Nat..

[CR30] Gu Y, Albuquerque CP, Braas D, Zhang W, Villa GR, Bi J (2017). mTORC2 regulates amino acid metabolism in Cancer by phosphorylation of the Cystine-glutamate Antiporter xCT. Mol Cell.

[CR31] Gupta S, Hau AM, Beach JR, Harwalker J, Mantuano E, Gonias SL (2013). Mammalian target of rapamycin complex 2 (mTORC2) is a critical determinant of bladder cancer invasion. PLoS One.

[CR32] Hagiwara A, Cornu M, Cybulski N, Polak P, Betz C, Trapani F (2012). Hepatic mTORC2 activates glycolysis and lipogenesis through Akt, glucokinase, and SREBP1c. Cell Metab.

[CR33] Han L, Wu JL, Yang LX (2012). Effect of combination of rapamycin and cisplatin on human cervical carcinoma Hela cells. Contemp Oncol (Pozn).

[CR34] Hanahan D, Weinberg RA (2011). Hallmarks of cancer: the next generation. Cell..

[CR35] Herbst RS, Morgensztern D, Boshoff C (2018). The biology and management of non-small cell lung cancer. Nat..

[CR36] Hirsch E, Ciraolo E, Franco I, Ghigo A, Martini M (2014). PI3K in cancer-stroma interactions: bad in seed and ugly in soil. Oncogene..

[CR37] Hu X, Wang Z, Chen M, Chen X, Liang W (2018). The anti-osteosarcoma cell activity by a mTORC1/2 dual inhibitor RES-529. Biochem Biophys Res Commun.

[CR38] Huang J, Wu S, Wu CL, Manning BD (2009). Signaling events downstream of mammalian target of rapamycin complex 2 are attenuated in cells and tumors deficient for the tuberous sclerosis complex tumor suppressors. Cancer Res.

[CR39] Im SJ, Hashimoto M, Gerner MY, Lee J, Kissick HT, Burger MC (2016). Defining CD8+ T cells that provide the proliferative burst after PD-1 therapy. Nat..

[CR40] Inaba K, Oda K, Ikeda Y, Sone K, Miyasaka A, Kashiyama T (2015). Antitumor activity of a combination of dual PI3K/mTOR inhibitor SAR245409 and selective MEK1/2 inhibitor pimasertib in endometrial carcinomas. Gynecol Oncol.

[CR41] Jiang WJ, Feng RX, Liu JT, Fan LL, Wang H, Sun GP (2017). RICTOR expression in esophageal squamous cell carcinoma and its clinical significance. Med Oncol.

[CR42] Julien LA, Carriere A, Moreau J, Roux PP (2010). mTORC1-activated S6K1 phosphorylates Rictor on threonine 1135 and regulates mTORC2 signaling. Mol Cell Biol.

[CR43] June CH, Warshauer JT, Bluestone JA (2017). Is autoimmunity the Achilles' heel of cancer immunotherapy?. Nat Med.

[CR44] Kamisawa T, Wood LD, Itoi T, Takaori K (2016). Pancreatic cancer. Lancet (London, England).

[CR45] Karin M (2006). Nuclear factor-kappaB in cancer development and progression. Nat..

[CR46] Kawata T, Tada K, Kobayashi M, Sakamoto T, Takiuchi Y, Iwai F (2018). Dual inhibition of the mTORC1 and mTORC2 signaling pathways is a promising therapeutic target for adult T-cell leukemia. Cancer Sci.

[CR47] Kim JO, Kim KH, Song IS, Cheon KS, Kim OH, Lee SC (2017). Potentiation of the anticancer effects of everolimus using a dual mTORC1/2 inhibitor in hepatocellular carcinoma cells. Oncotarget..

[CR48] Kim ST, Kim SY, Klempner SJ, Yoon J, Kim N, Ahn S (2017). Rapamycin-insensitive companion of mTOR (RICTOR) amplification defines a subset of advanced gastric cancer and is sensitive to AZD2014-mediated mTORC1/2 inhibition. Ann Oncol.

[CR49] Kimura K, Huang RC (2016). Tetra-O-methyl Nordihydroguaiaretic acid broadly suppresses Cancer metabolism and synergistically induces strong anticancer activity in combination with Etoposide, Rapamycin and UCN-01. PLoS One.

[CR50] Knight SD, Adams ND, Burgess JL, Chaudhari AM, Darcy MG, Donatelli CA (2010). Discovery of GSK2126458, a highly potent inhibitor of PI3K and the mammalian target of Rapamycin. ACS Med Chem Lett.

[CR51] Lampada A, O'Prey J, Szabadkai G, Ryan KM, Hochhauser D, Salomoni P (2017). mTORC1-independent autophagy regulates receptor tyrosine kinase phosphorylation in colorectal cancer cells via an mTORC2-mediated mechanism. Cell Death Differ.

[CR52] Lemmon MA, Schlessinger J (2010). Cell signaling by receptor tyrosine kinases. Cell..

[CR53] Li X, Gao T (2014). mTORC2 phosphorylates protein kinase Czeta to regulate its stability and activity. EMBO Rep.

[CR54] Li X, Li Z, Song Y, Liu W, Liu Z (2018). The mTOR kinase inhibitor CZ415 inhibits human papillary thyroid carcinoma cell growth. Cell Physiol Biochem.

[CR55] Lin XM, Hu L, Gu J, Wang RY, Li L, Tang J (2017). Choline kinase alpha mediates interactions between the epidermal growth factor receptor and mechanistic target of Rapamycin complex 2 in hepatocellular carcinoma cells to promote drug resistance and Xenograft tumor progression. Gastroenterol..

[CR56] Liu ZG, Tang J, Chen Z, Zhang H, Wang H, Yang J (2016). The novel mTORC1/2 dual inhibitor INK128 enhances radiosensitivity of breast cancer cell line MCF-7. Int J Oncol.

[CR57] Luchman HA, Stechishin OD, Nguyen SA, Lun XQ, Cairncross JG, Weiss S (2014). Dual mTORC1/2 blockade inhibits glioblastoma brain tumor initiating cells in vitro and in vivo and synergizes with temozolomide to increase orthotopic xenograft survival. Clin Cancer Res.

[CR58] Martel RR, Klicius J, Galet S (1977). Inhibition of the immune response by rapamycin, a new antifungal antibiotic. Can J Physiol Pharmacol.

[CR59] Masui K, Tanaka K, Akhavan D, Babic I, Gini B, Matsutani T (2013). mTOR complex 2 controls glycolytic metabolism in glioblastoma through FoxO acetylation and upregulation of c-Myc. Cell Metab.

[CR60] Masui K, Tanaka K, Ikegami S, Villa GR, Yang H, Yong WH (2015). Glucose-dependent acetylation of Rictor promotes targeted cancer therapy resistance. Proc Natl Acad Sci U S A.

[CR61] Mecca C, Giambanco I, Bruscoli S, Bereshchenko O, Fioretti B, Riccardi C (2018). PP242 counteracts Glioblastoma cell proliferation, migration, invasiveness and Stemness properties by inhibiting mTORC2/AKT. Front Cell Neurosci.

[CR62] Miller TW, Balko JM, Arteaga CL (2011). Phosphatidylinositol 3-kinase and antiestrogen resistance in breast cancer. J Clin Oncol.

[CR63] Miller TW, Rexer BN, Garrett JT, Arteaga CL (2011). Mutations in the phosphatidylinositol 3-kinase pathway: role in tumor progression and therapeutic implications in breast cancer. Breast Cancer Res.

[CR64] Milosevic Z, Bankovic J, Dinic J, Tsimplouli C, Sereti E, Dragoj M (2018). Potential of the dual mTOR kinase inhibitor AZD2014 to overcome paclitaxel resistance in anaplastic thyroid carcinoma. Cell Oncol (Dordrecht).

[CR65] Molina JR, Yang P, Cassivi SD, Schild SE, Adjei AA (2008). Non-small cell lung cancer: epidemiology, risk factors, treatment, and survivorship. Mayo Clin Proc.

[CR66] Morrison Joly M, Hicks DJ, Jones B, Sanchez V, Estrada MV, Young C (2016). Rictor/mTORC2 drives progression and therapeutic resistance of HER2-amplified breast cancers. Cancer Res.

[CR67] Morrison Joly M, Williams MM, Hicks DJ, Jones B, Sanchez V, Young CD (2017). Two distinct mTORC2-dependent pathways converge on Rac1 to drive breast cancer metastasis. Breast Cancer Res.

[CR68] Motzer RJ, Hutson TE, Ren M, Dutcus C, Larkin J (2016). Independent assessment of lenvatinib plus everolimus in patients with metastatic renal cell carcinoma. Lancet Oncol.

[CR69] Musa F, Alard A, David-West G, Curtin JP, Blank SV, Schneider RJ (2016). Dual mTORC1/2 inhibition as a novel strategy for the Resensitization and treatment of platinum-resistant ovarian Cancer. Mol Cancer Ther.

[CR70] Oh WJ, Jacinto E (2011). mTOR complex 2 signaling and functions. Cell Cycle.

[CR71] O'Reilly KE, Rojo F, She QB, Solit D, Mills GB, Smith D (2006). mTOR inhibition induces upstream receptor tyrosine kinase signaling and activates Akt. Cancer Res.

[CR72] Palm W, Park Y, Wright K, Pavlova NN, Tuveson DA, Thompson CB (2015). The utilization of extracellular proteins as nutrients is suppressed by mTORC1. Cell..

[CR73] Pan XD, Gu DH, Mao JH, Zhu H, Chen X, Zheng B (2017). Concurrent inhibition of mTORC1 and mTORC2 by WYE-687 inhibits renal cell carcinoma cell growth in vitro and in vivo. PLoS One.

[CR74] Pao W, Hutchinson KE (2012). Chipping away at the lung cancer genome. Nat Med.

[CR75] Plas DR, Thompson CB (2005). Akt-dependent transformation: there is more to growth than just surviving. Oncogene..

[CR76] Pollizzi KN, Patel CH, Sun IH, Oh MH, Waickman AT, Wen J (2015). mTORC1 and mTORC2 selectively regulate CD8(+) T cell differentiation. J Clin Invest.

[CR77] Raich-Regue D, Rosborough BR, Watson AR, McGeachy MJ, Turnquist HR, Thomson AW (2015). mTORC2 Deficiency in Myeloid Dendritic Cells Enhances Their Allogeneic Th1 and Th17 Stimulatory Ability after TLR4 Ligation In Vitro and In Vivo. J Immunol.

[CR78] Reardon DA, Desjardins A, Vredenburgh JJ, Gururangan S, Friedman AH, Herndon JE (2010). Phase 2 trial of erlotinib plus sirolimus in adults with recurrent glioblastoma. J Neuro Oncol.

[CR79] Robinson DR, Wu YM, Lin SF (2000). The protein tyrosine kinase family of the human genome. Oncogene..

[CR80] Rozengurt E, Soares HP, Sinnet-Smith J (2014). Suppression of feedback loops mediated by PI3K/mTOR induces multiple overactivation of compensatory pathways: an unintended consequence leading to drug resistance. Mol Cancer Ther.

[CR81] Ruder D, Papadimitrakopoulou V, Shien K, Behrens C, Kalhor N, Chen H (2018). Concomitant targeting of the mTOR/MAPK pathways: novel therapeutic strategy in subsets of RICTOR/KRAS-altered non-small cell lung cancer. Oncotarget..

[CR82] Sadowski SM, Boufraqech M, Zhang L, Mehta A, Kapur P, Zhang Y (2015). Torin2 targets dysregulated pathways in anaplastic thyroid cancer and inhibits tumor growth and metastasis. Oncotarget..

[CR83] Sakre N, Wildey G, Behtaj M, Kresak A, Yang M, Fu P (2017). RICTOR amplification identifies a subgroup in small cell lung cancer and predicts response to drugs targeting mTOR. Oncotarget..

[CR84] Sarbassov DD, Guertin DA, Ali SM, Sabatini DM (2005). Phosphorylation and regulation of Akt/PKB by the rictor-mTOR complex. Sci..

[CR85] Saxton RA, Sabatini DM (2017). mTOR signaling in growth, metabolism, and disease. Cell..

[CR86] Schmidt KM, Hellerbrand C, Ruemmele P, Michalski CW, Kong B, Kroemer A (2017). Inhibition of mTORC2 component RICTOR impairs tumor growth in pancreatic cancer models. Oncotarget..

[CR87] Shafer A, Zhou C, Gehrig PA, Boggess JF, Bae-Jump VL (2010). Rapamycin potentiates the effects of paclitaxel in endometrial cancer cells through inhibition of cell proliferation and induction of apoptosis. Int J Cancer.

[CR88] Sharma P, Allison JP (2015). The future of immune checkpoint therapy. Sci (New York, NY).

[CR89] Shi F, Zhang J, Liu H, Wu L, Jiang H, Wu Q (2018). The dual PI3K/mTOR inhibitor dactolisib elicits anti-tumor activity in vitro and in vivo. Oncotarget..

[CR90] Shuhua W, Chenbo S, Yangyang L, Xiangqian G, Shuang H, Tangyue L (2015). Autophagy-related genes Raptor, Rictor, and Beclin1 expression and relationship with multidrug resistance in colorectal carcinoma. Hum Pathol.

[CR91] Siegel RL, Miller KD, Jemal A (2017). Cancer statistics, 2017. CA Cancer J Clin.

[CR92] Siegel RL, Miller KD, Jemal A (2019). Cancer statistics, 2019. CA Cancer J Clin.

[CR93] Tan AC, Vyse S, Huang PH (2017). Exploiting receptor tyrosine kinase co-activation for cancer therapy. Drug Discov Today.

[CR94] Tanaka K, Babic I, Nathanson D, Akhavan D, Guo D, Gini B (2011). Oncogenic EGFR signaling activates an mTORC2-NF-kappaB pathway that promotes chemotherapy resistance. Cancer Discov..

[CR95] Torki S, Soltani A, Shirzad H, Esmaeil N, Ghatrehsamani M (2017). Synergistic antitumor effect of NVP-BEZ235 and CAPE on MDA-MB-231 breast cancer cells. Biomed Pharmacother.

[CR96] Venkatesan AM, Dehnhardt CM, Delos Santos E, Chen Z, Dos Santos O, Ayral-Kaloustian S (2010). Bis (morpholino-1,3,5-triazine) derivatives: potent adenosine 5′-triphosphate competitive phosphatidylinositol-3-kinase/mammalian target of rapamycin inhibitors: discovery of compound 26 (PKI-587), a highly efficacious dual inhibitor. J Med Chem.

[CR97] Waks AG, Winer EP (2019). Breast Cancer treatment: a review. Jama..

[CR98] Waldner M, Fantus D, Solari M, Thomson AW (2016). New perspectives on mTOR inhibitors (rapamycin, rapalogs and TORKinibs) in transplantation. Br J Clin Pharmacol.

[CR99] Wang F, Meng M, Mo B, Yang Y, Ji Y, Huang P (2018). Crosstalks between mTORC1 and mTORC2 variagate cytokine signaling to control NK maturation and effector function. Nat Commun.

[CR100] Wang JY, Jin X, Zhang X, Li XF (2018). CC-223 inhibits human head and neck squamous cell carcinoma cell growth. Biochem Biophys Res Commun.

[CR101] Wang L, Qi J, Yu J, Chen H, Zou Z, Lin X (2017). Overexpression of Rictor protein in colorectal cancer is correlated with tumor progression and prognosis. Oncol Lett.

[CR102] Wang L, Zhu YR, Wang S, Zhao S (2016). Autophagy inhibition sensitizes WYE-354-induced anti-colon cancer activity in vitro and in vivo. Tumour Biol.

[CR103] Watanabe T, Sato A, Kobayashi-Watanabe N, Sueoka-Aragane N, Kimura S, Sueoka E (2016). Torin2 potentiates anticancer effects on adult T-cell leukemia/lymphoma by inhibiting mammalian target of Rapamycin. Anticancer Res.

[CR104] Weber H, Leal P, Stein S, Kunkel H, Garcia P, Bizama C (2015). Rapamycin and WYE-354 suppress human gallbladder cancer xenografts in mice. Oncotarget..

[CR105] Werfel TA, Wang S, Jackson MA, Kavanaugh TE, Joly MM, Lee LH (2018). Selective mTORC2 inhibitor therapeutically blocks breast Cancer cell growth and survival. Cancer Res.

[CR106] Wong Te Fong AC, Thavasu P, Gagrica S, Swales KE, Leach MO, Cosulich SC (2017). Evaluation of the combination of the dual m-TORC1/2 inhibitor vistusertib (AZD2014) and paclitaxel in ovarian cancer models. Oncotarget..

[CR107] Xie J, Li Q, Ding X, Gao Y (2017). GSK1059615 kills head and neck squamous cell carcinoma cells possibly via activating mitochondrial programmed necrosis pathway. Oncotarget..

[CR108] Xie J, Li Q, Ding X, Gao Y (2018). Targeting mTOR by CZ415 inhibits head and neck squamous cell carcinoma cells. Cell Physiol Biochem.

[CR109] Xie Z, Wang J, Liu M, Chen D, Qiu C, Sun K (2017). CC-223 blocks mTORC1/C2 activation and inhibits human hepatocellular carcinoma cells in vitro and in vivo. PLoS One.

[CR110] Xu AM, Huang PH (2010). Receptor tyrosine kinase coactivation networks in cancer. Cancer Res.

[CR111] Xu DQ, Toyoda H, Yuan XJ, Qi L, Chelakkot VS, Morimoto M (2018). Anti-tumor effect of AZD8055 against neuroblastoma cells in vitro and in vivo. Exp Cell Res.

[CR112] Xue Q, Hopkins B, Perruzzi C, Udayakumar D, Sherris D, Benjamin LE (2008). Palomid 529, a novel small-molecule drug, is a TORC1/TORC2 inhibitor that reduces tumor growth, tumor angiogenesis, and vascular permeability. Cancer Res.

[CR113] Yamaoka Toshimitsu, Kusumoto Sojiro, Ando Koichi, Ohba Motoi, Ohmori Tohru (2018). Receptor Tyrosine Kinase-Targeted Cancer Therapy. International Journal of Molecular Sciences.

[CR114] Yang G, Murashige DS, Humphrey SJ, James DE (2015). A positive feedback loop between Akt and mTORC2 via SIN1 phosphorylation. Cell Rep.

[CR115] Yu G, Wang J, Chen Y, Wang X, Pan J, Li G (2009). Overexpression of phosphorylated mammalian target of rapamycin predicts lymph node metastasis and prognosis of chinese patients with gastric cancer. Clin Cancer Res.

[CR116] Yu P, Laird AD, Du X, Wu J, Won KA, Yamaguchi K (2014). Characterization of the activity of the PI3K/mTOR inhibitor XL765 (SAR245409) in tumor models with diverse genetic alterations affecting the PI3K pathway. Mol Cancer Ther.

[CR117] Yu Y, Yu X, Ma J, Tong Y, Yao J (2016). Effects of NVP-BEZ235 on the proliferation, migration, apoptosis and autophagy in HT-29 human colorectal adenocarcinoma cells. Int J Oncol.

[CR118] Zaidi AH, Kosovec JE, Matsui D, Omstead AN, Raj M, Rao RR (2017). PI3K/mTOR dual inhibitor, LY3023414, demonstrates potent antitumor efficacy against esophageal adenocarcinoma in a rat model. Ann Surg.

[CR119] Zeng H (2017). mTOR signaling in immune cells and its implications for cancer immunotherapy. Cancer Lett.

[CR120] Zhang F, Zhang X, Li M, Chen P, Zhang B, Guo H (2010). mTOR complex component Rictor interacts with PKCzeta and regulates cancer cell metastasis. Cancer Res.

[CR121] Zhang H, Dou J, Yu Y, Zhao Y, Fan Y, Cheng J (2015). mTOR ATP-competitive inhibitor INK128 inhibits neuroblastoma growth via blocking mTORC signaling. Apoptosis.

[CR122] Zhang L, Tschumi BO, Lopez-Mejia IC, Oberle SG, Meyer M, Samson G (2016). Mammalian target of Rapamycin complex 2 controls CD8 T cell memory differentiation in a Foxo1-dependent manner. Cell Rep.

[CR123] Zhang S, Song X, Cao D, Xu Z, Fan B, Che L (2017). Pan-mTOR inhibitor MLN0128 is effective against intrahepatic cholangiocarcinoma in mice. J Hepatol.

[CR124] Zheng G, Jia X, Peng C, Deng Y, Yin J, Zhang Z (2015). The miR-491-3p/mTORC2/FOXO1 regulatory loop modulates chemo-sensitivity in human tongue cancer. Oncotarget..

[CR125] Zhi X, Chen W, Xue F, Liang C, Chen BW, Zhou Y (2015). OSI-027 inhibits pancreatic ductal adenocarcinoma cell proliferation and enhances the therapeutic effect of gemcitabine both in vitro and in vivo. Oncotarget..

[CR126] Zhou HY, Huang SL (2012). Current development of the second generation of mTOR inhibitors as anticancer agents. Chin J Cancer.

